# How to approximate fuzzy sets: mind-changes and the Ershov Hierarchy

**DOI:** 10.1007/s11229-023-04056-y

**Published:** 2023-02-06

**Authors:** Nikolay Bazhenov, Manat Mustafa, Sergei Ospichev, Luca San Mauro

**Affiliations:** 1 Sobolev Institute of Mathematics, 4 Acad. Koptyug Ave., Novosibirsk, Russia 630090; 2 Department of Mathematics, School of Sciences and Humanities, Nazarbayev University, 53 Qabanbaybatyr Avenue, 010000 Astana, Kazakhstan; 3 Institute of Discrete Mathematics and Geometry, Vienna University of Technology, Wiedner Hauptstraße 8-10/104, 1040 Vienna, Austria

**Keywords:** Fuzzy set, Computability theory, *n*-Computably enumerable set, Ershov Hierarchy, 03E72, 03D55

## Abstract

Computability theorists have introduced multiple hierarchies to measure the complexity of sets of natural numbers. The Kleene Hierarchy classifies sets according to the first-order complexity of their defining formulas. The Ershov Hierarchy classifies limit computable sets with respect to the number of mistakes that are needed to approximate them. Biacino and Gerla extended the Kleene Hierarchy to the realm of fuzzy sets, whose membership functions range in a complete lattice. In this paper, we combine the Ershov Hierarchy and fuzzy set theory, by introducing and investigating the Fuzzy Ershov Hierarchy.

## Introduction

Suppose you wish to know *exactly* whether a certain object satisfies some property $\mathcal {P}$. Two sorts of difficulties may arise:

1. $\mathcal {P}$ may be graded, that is, some objects satisfy $\mathcal {P}$ only *up to some degree*;
2. Or rather, membership to $\mathcal {P}$ may be sharply defined but knowing whether an arbitrary object satisfies $\mathcal {P}$ may exceed the capabilities of any computer—or, equivalently, of any human armed with pencil, paper, and endless patience.

The first case is studied in fuzzy mathematics; the second one in computability theory. In this work, we’ll discuss a natural way of merging these approaches.

*Crisp properties* on a given domain $\mathcal {D}$—i.e., properties whose membership functions range in the set $\{0,1\}$—can be naturally identified with subsets of $\mathcal {D}$. By adopting this perspective, one may regard classical computability theory as the study of the complexity of crisp properties on the set $\omega$ of the natural numbers: e.g., “being even” and “being the code of a Turing machine which halts on a blank tape” are examples of, respectively, a decidable crisp property and an undecidable one.

Computability theorists have introduced multiple hierarchies to measure the complexity of crisp properties. Two such hierarchies will be relevant for the present paper. The Kleene Hierarchy classifies subsets of $\omega$ according to the first-order complexity of their defining formulas within arithmetic. The Ershov Hierarchy concentrates on an important initial segment of the Kleene Hierarchy, that of $\Delta ^0_2$ sets (which coincide with the sets that are computable in the limit), by classifying such sets with respect to the number of mistakes that are needed to approximate them.

*Fuzzy sets*, introduced by Zadeh (1965) and later developed into a broad area of research, allow to mathematically study *graded properties*, such as those properties with blurry boundaries, and to extend the scope of logic to *approximate reasoning*.

It is natural to ask how to introduce computability theory within fuzzy mathematics. A first approach is to define fuzzy algorithms (as in, e.g., Bedregal and Figueira (2006), Santos (1970), Wiedermann (2002), and Zadeh (1968)), and then rebuild computability theory by permitting fuzzy computations. A parallel approach is to maintain ordinary Turing machines and just adopt them to calibrate the complexity of fuzzy sets. After all, well-established computability-theoretic hierarchies could be extended to the realm of fuzzy objects. This is the case for the Kleene Hierarchy, which has been extended to fuzzy sets by Biacino and Gerla (1989), see also Gerla (2001), Harkleroad (1984), and Harkleroad (1988).

In this paper, we introduce and investigate the Fuzzy Ershov Hierarchy (**FEH**). That is, we focus on the complexity of approximating fuzzy objects which belong to the class $\Delta ^0_2$. The key idea for evaluating this complexity is that of a *mind-change*, which is borrowed from Ershov (1968a, b, 1970) (and it has been intensively studied—see, e.g., Bazhenov et al. (2020), Cooper et al. (1991), Downey and Greenberg (2020), and Stephan et al. (2009)). Intuitively, mind-changes allow to improve knowledge of a given property $\mathcal {P}$ through time. For example, suppose that $\mathcal {P}$ is the following property: “being a theorem of Peano Arithmetic”. Such a property is certainly crisp but, as is well-known, is also undecidable—that is to say, there can be no algorithm which, for all arithmetic formulas $\varphi$, can determine, in finitely many steps, whether Peano Arithmetic proves $\varphi$ or not. Yet, an ideal agent $\textbf{A}$, which is allowed to change its mind, can eventually achieve knowledge of $\mathcal {P}$ as follows:

- At first, $\textbf{A}$ believes that only the axioms of Peano Arithmetic satisfy $\mathcal {P}$;
- Next, $\textbf{A}$ lists, one-by-one, all the consequences of such axioms;
- Finally, whenever a formula $\varphi$ appears in the above list, $\textbf{A}$ changes its mind about the theoremhood of $\varphi$, by declaring that $\varphi$ does satisfy $\mathcal {P}$.

Hence, following this procedure, $\textbf{A}$ can gradually increase knowledge of $\mathcal {P}$ in such a way that, in the limit, $\textbf{A}$ will correctly guess the theoremhood of all arithmetic formulas, and for any such formula at most one mind-change will be required.

In the classical setting, an approximation to a set *A* changes its mind on a given input *x* by switching its guess on whether *x* belongs to *A* or not. Moving to fuzzy sets, mind-changes will be formalized by changes in the monotonicity of approximating functions. We will prove that, by allowing more and more mind-changes, we will be able to capture larger and larger sub-classes of $\Delta ^0_2$ fuzzy sets. In particular, it will follow that there are fuzzy sets which cannot be approximated only from above or below, but they require approximations which oscillate “up and down” on the membership degree of *x*, for some inputs *x*.

The paper is arranged as follows. In Sect. 2, we recall preliminaries concerning fuzzy sets, effective reals, and the Ershov Hierarchy. In Sect. 3, we introduce **FEH**, and we prove some of the main results of the paper. First, the hierarchy does not collapse (Proposition 5). Second, in analogy with the classical case, sets lying at the so-called finite levels of **FEH** can be represented as Boolean combinations of fuzzy sets belonging to the first level, i.e., c.e. fuzzy sets (Theorem 6). Third, contrary to the classical case, **FEH** does not exhaust the class of all $\Delta ^0_2$ fuzzy sets (Proposition 8). In Sect. 4, we investigate two natural ways of broadening **FEH**. First, we refine the proposed hierarchy, by keeping track of all updates needed to approximate a $\Delta ^0_2$ fuzzy set, rather than focusing exclusively on those updates which determine a change of monotonicity in the approximating function. We show that such a refined hierarchy is quite wild (Theorem 10). Second, in analogy with the Classical Ershov Hierarchy, we extend **FEH** to the transfinite. Yet, in sharp contrast with the classical case, we note that even including all transfinite levels, we still do not exhaust all $\Delta ^0_2$ fuzzy sets.

## Preliminaries

We assume that the reader is familiar with the basic notions of computability theory. For the background, we refer to the monographs (Rogers, 1967; Soare, 2016). Anyway, let us include here definitions of the most basic notions:

- A (partial) function $f: \omega \rightarrow \omega$ is *computable* if there is a Turing machine $\textbf{M}_f$ so that, for all natural numbers *x* and *y*, $f(x)=y$ holds if and only if $\textbf{M}_f$, having a suitable coding of *x* printed on its input tape, halts after finitely many steps with a suitable coding of *y* printed on its output tape;
- A (crisp) set $A\subseteq \omega$ is *computable* if its characteristic function, defined by
  $$\begin{aligned} \chi _A(x):={\left\{ \begin{array}{ll} 1 &{}x\in A,\\ 0 &{}x\notin A, \end{array}\right. } \end{aligned}$$
  is computable.
- A (crisp) set $A\subseteq \omega$ is *computably enumerable* (c.e.) if it is the image of a computable function. Equivalently, c.e. sets can be seen as those sets which are the domain of some computable function.

Note that, as is now custom in computability theory, this paper uses the term *computably enumerable* (or *c.e.*) in place of *recursively enumerable*. For a set *X*, by $\vert X \vert$ we denote the cardinality of *X*.

The preliminaries on fuzzy sets mainly follow Gerla (2001). As usual, one fixes an effective bijection $\nu :\mathbb {Q}\rightarrow \omega$. This convention allows to transfer familiar computability-theoretic notions to the rationals: for example, a crisp set $X\subseteq \mathbb {Q}$ is computable iff $\nu (X)$ is a computable subset of $\omega$.

### Fuzzy subsets

Let $\mathcal {L}$ be a complete lattice. A *fuzzy subset* (or an $\mathcal {L}$-subset) of $\omega$ is an arbitrary function $A :\omega \rightarrow \mathcal {L}$. In this paper, for the sake of simplicity, we consider the case when $\mathcal {L}$ is equal to the real interval $[0,1]_{\mathbb {R}}$. A fuzzy subset *A* is *crisp* if $A(x) \in \{ 0,1\}$ for all $x\in \omega$.

As mentioned in the introduction, a fundamental tool for classifying the complexity of crisp subsets of $\omega$ is provided by the Kleene Arithmetical Hierarchy (Kleene, 1943) (see, e.g., Chapter 4 in Soare (2016) for a detailed discussion). Biacino and Gerla (1989) extended the Kleene Hierarchy to fuzzy subsets. In our paper, we work only with fuzzy subsets belonging to the levels $\Sigma ^0_1$, $\Pi ^0_1$, and $\Delta ^0_2$ of the Kleene Hierarchy. Hence, we give formal definitions only for these levels. For more details, the reader is referred to Biacino and Gerla (1989) and Sect. 11.5 in Gerla (2001). By $[0,1]_{\mathbb {Q}}$ we denote the set of all rational numbers *q* such that $0\le q \le 1$.

#### Definition 1

((Biacino & Gerla, 1989), see also Sect. 11.2 in Gerla (2001)) A fuzzy set *A* is *computably enumerable* (or belongs to the class $\Sigma ^0_1$) if there is a computable function $f:\omega \times \omega \rightarrow [0,1]_{\mathbb {Q}}$ such that, for all $x\in \omega$, we have:

- $$\lim _{s\rightarrow \infty }f(x,s)=A(x)$$;
- $$(\forall s)(f(x,s+1)\ge f(x,s))$$.

We say that such function *f* is a $\Sigma ^0_1$-*approximation* of the fuzzy set *A*.

Note that without loss of generality, one can always assume that in the definition above, *f*(*x*, 0) equals 0. Hence, c.e. fuzzy sets may intuitively be regarded as fuzzy sets which can be approximated “from below”, in the sense that approximations to c.e. fuzzy sets can only increase over time.

If *A* and *B* are fuzzy sets, then one can define set-theoretic operations on them:

- Union: $$(A\cup B)(x) = \max \{ A(x), B(x)\}$$.
- Intersection: $$(A\cap B)(x) = \min \{A(x), B(x)\}$$.
- Complement: $$\overline{A}(x) = 1 - A(x)$$.

A fuzzy set *A* is *co-computably enumerable* (or belongs to the class $\Pi ^0_1$) if its complement $\overline{A}$ is c.e. Equivalently (see Theorem 5.2 in Gerla (2001), Chap. 11), *A* is co-c.e. if and only if there is a computable function $f:\omega ^2 \rightarrow [0,1]_{\mathbb {Q}}$ such that, for all $x\in \omega$, we have:

(*a*): $$\lim _{s\rightarrow \infty } f(x,s)=A(x)$$;
(*b*$$'$$)
$$(\forall s)(f(x,s+1)\le f(x,s))$$.

In the $\Pi ^0_1$ case, we may assume that $f(x,0)=1$, for all *x*. So, co-c.e. fuzzy sets may be regarded as fuzzy sets which can be approximated “from above”.

Finally, the main object of study of this paper are $\Delta ^0_2$ fuzzy sets. A fuzzy set *A* *belongs to the class* $\Delta ^0_2$ if *A* lies in both classes $\Sigma ^0_2$ and $\Pi ^0_2$ of the Kleene Hierarchy. In this paper, we adopt the following equivalent definition (see Proposition 5.4 in Gerla (2001), Chap. 11).

#### Definition 2

A fuzzy set *A* is $\Delta ^0_2$ if and only if there is a computable function $f:\omega ^2 \rightarrow [0,1]_{\mathbb {Q}}$ such that $\lim _{s\rightarrow \infty }f(x,s)=A(x)$, for all $x\in \omega$. We call such function *f*(*x*, *s*) a $\Delta ^0_2$-*approximation* of the fuzzy set *A*.

### Effective reals

Here we briefly discuss some simple results which connect fuzzy subsets of $\omega$ with effectively approximable reals. Informally speaking, a real $\alpha \in \mathbb {R}$ is effectively approximable if there exists an algorithm which provides an approximation of $\alpha$ (typically, one can view an approximation as a sequence of rational numbers). One of the first mathematical formalizations of this concept was given by Turing (1936): he defined computable real numbers as those reals that have a binary expansion which can be computed by a Turing machine. Computability theorists also consider weaker notions of effective approximability—for example, left-c.e. reals are precisely those reals $\alpha$ such that the set of all rationals $q<\alpha$ can be enumerated by a Turing machine (a more formal definition of a left-c.e. real is given below). We refer to Chapter 5 in Downey and Hirschfeldt (2010) for further details.

We consider reals $\alpha \in [0,1]_{\mathbb {R}}$. A real $\alpha$ is *left-c.e.* if the set $\{ q\in \mathbb {Q}\,:q < \alpha \}$ is c.e; $\alpha$ is *right-c.e.* if the set $\{ q\in \mathbb {Q} \,:q > \alpha \}$ is c.e. Moreover, a countable sequence $(\alpha _k)_{k\in \omega }$ is *uniformly left-c.e.* if the set

$$\begin{aligned} \{ (k,q) \in \omega \times \mathbb {Q} \,:q < \alpha _k\} \end{aligned}$$

is c.e.; $(\alpha _k)_{k\in \omega }$ is *uniformly right-c.e.* if the set

$$\begin{aligned} \{ (k,q) \in \omega \times \mathbb {Q} \,:q > \alpha _k\} \end{aligned}$$

is c.e. By working with these definitions, it is not hard to prove the following result.

#### Proposition 1

Let *A* be a fuzzy subset of $\omega$.

1. *A* is c.e. if and only if the reals *A*(*k*), $k\in \omega$, are uniformly left-c.e.
2. *A* is co-c.e. if and only if the reals *A*(*k*), $k\in \omega$, are uniformly right-c.e.

A real $\alpha$ is $\Delta ^0_2$ if there is a computable sequence $(q_s)_{s\in \omega }$ of rationals such that $\alpha = \lim _{s\rightarrow \infty } q_s$ (see, e.g., Theorem 5.1.3 in Downey and Hirschfeldt (2010)). We also observe the following, which is an immediate consequence of the definitions:

#### Proposition 2

A fuzzy set *A* is $\Delta ^0_2$ if and only if the reals *A*(*k*), $k\in \omega$, are uniformly $\Delta ^0_2$, i.e., there is a computable sequence $(q_{k,s})_{k,s\in \omega }$ of rationals such that $A(k) = \lim _{s\rightarrow \infty } q_{k,s}$, for all *k*.

### The Classical Ershov Hierarchy

We give few preliminaries on the Ershov (1968a, b, 1970); to distinguish it from the fuzzy analogue introduced below, we refer to this hierarchy as the Classical Ershov Hierarchy. For the sake of simplicity, here we discuss only the finite levels of the hierarchy (these finite levels are also called *Difference Hierarchy* in the literature). In this section, all subsets of $\omega$ are crisp.

#### Definition 3

Let $n\ge 1$. A set $A\subseteq \omega$ is *n*-*computably enumerable* (or *n*-*c.e.*, or belongs to the class $\Sigma ^{-1}_n$) if there is a computable function $f:\omega \times \omega \rightarrow \{ 0,1\}$ such that for all $x\in \omega$, we have

- $$\lim _{s\rightarrow \infty } f(x,s) = A(x)$$;
- $$f(x,0) = 0$$;
- $$ \vert \{ s \,:f(x,s) \ne f(x,s+1)\}\vert \le n$$.

A set *A* is *co-n*-*computably enumerable* (or *co*-*n*-*c.e.*, or belongs to the class $\Pi ^{-1}_n$) if its complement $\overline{A}$ is *n*-c.e.

#### Remark 1

It is common to refer to the numbers labeled by the variable *s* in the last definition as *stages*. This reflects the intuition that *f* is a procedure to approximate *A* through time (that is, by stages). So, in plain terms, a set *A* is *n*-c.e. if one is eventually able to achieve complete knowledge of *A* by some stage-by-stage procedure which, for each number *x*, has at most *n* many mind-changes as to whether the property “*x* belongs to *A*” holds or not.

Historically, the notion of *n*-c.e. sets was introduced by Putnam (1965) and Gold (1965). Note that

$$\begin{aligned} \Sigma ^{-1}_n \cup \Pi ^{-1}_n \subseteq \Sigma ^{-1}_{n+1} \cap \Pi ^{-1}_{n+1}. \end{aligned}$$

Ershov (1968a) proved that, for each $n\ge 1$, there exists an *n*-c.e. set $S_n$ such that every *n*-c.e. set *A* is many-one reducible to $S_n$ (i.e., there exists a computable function *f* so that

$$\begin{aligned} x \in A \Leftrightarrow f(x) \in S_n, \end{aligned}$$

for all $x\in \omega$). In addition, $S_n$ does not belong to $\Pi ^{-1}_n$. In particular, this implies that the Classical Ershov Hierarchy does not collapse.

Sets from the class $\Sigma ^{-1}_n$ can be represented as Boolean combinations of c.e. sets:

#### Theorem 3

(Ershov, 1968a) Let *k* be a natural number. A set $A\subseteq \omega$ is $(2k+1)$-c.e. if and only if there are c.e. sets $W_1,W_2,\dots ,W_{2k-1},W_{2k},W_{2k+1}$ such that

$$\begin{aligned} A = (W_1 \setminus W_2) \cup (W_3 \setminus W_4) \cup \dots \cup (W_{2k-1}\setminus W_{2k}) \cup W_{2k+1}. \end{aligned}$$

A set *A* is $(2k+2)$-c.e. if and only if there are c.e. sets $W_1,W_2,\dots ,W_{2k+1},W_{2k+2}$ such that

$$\begin{aligned} A = (W_1 \setminus W_2) \cup (W_3 \setminus W_4) \cup \dots \cup (W_{2k-1}\setminus W_{2k}) \cup (W_{2k+1}\setminus W_{2k+2}). \end{aligned}$$

We refer the reader to the survey (Stephan et al., 2009) for more details on the Classical Ershov Hierarchy.

## Fuzzy Ershov Hierarchy

In this section, we extend the classical Difference Hierarchy to the class of fuzzy subsets of $\omega$ (see Definition 5 below). We establish some initial properties of this hierarchy: the hierarchy does not collapse (Sect. 3.1); it is connected to the Boolean combinations of c.e. fuzzy sets (Sect. 3.2); the introduced levels of the hierarchy do not exhaust all $\Delta ^0_2$ fuzzy sets (Sect. 3.3).

As discussed in Remark 1, the Difference Hierarchy classifies $\Delta ^0_2$ sets according to the number of mind-changes that are needed to reliably approximate them. Now, note that mind-changes are naturally associated to changes of monotocity of the function *f*. Indeed, suppose that, for some input *x* and stage *s*, we have the following mind-change: $f(x,s)=0$ but $f(x,s+1)=1$ so that, on this input, *f* *increases* its output from 0 to 1. Then, in order to witness some further mind-change, there must exist a least stage $t>s$ at which *f*, on the same input, *decreases* from 1 to 0, thus switching its monotonicity. In order to formally define the Fuzzy Ershov Hierarchy (**FEH**), we will rely on this connection between mind-changes and changes of monotocity of the approximating function.

We begin by illustrating the intuition behind Definition 5 with the following example. A $\Delta ^0_2$ fuzzy set *A* is called 3-computably enumerable if it possesses a $\Delta ^0_2$-approximation *f*(*x*, *s*), which changes its mind *at most two times*. So, for an element $x\in \omega$, the worst case behavior looks like this:

- First, our approximation (non-strictly) increases—i.e., there is a stage $s_1$ such that $f(x,s) \le f(x,s+1)$, for all $s< s_1$.
- Second, the approximation starts to decrease until some stage $s_2>s_1$: $f(x, s_1) > f(x, s_1 +1)$ and $f(x,s) \ge f(x,s+1)$, for $s_1< s < s_2$.
- Then the final change of mind happens: the approximation will forever increase—$f(x,s_2) < f(x,s_2+1)$ and $f(x,s) \le f(x,s+1)$ for all $s> s_2$.

In order to make this idea formal, we introduce *mind-change functions*, which “track down” the described mind-changes.

### Definition 4

Let *f* be a total function from $\omega \times \omega$ to $[0,1]_{\mathbb {Q}}$. Its $\Sigma$-*mind-change function* $m^{f}_{\Sigma } :\omega \times \omega \rightarrow \{ -1, 1\}$ is defined as follows.

1. $$m^f_{\Sigma }(x,0) = 1$$.
2. Suppose that $$m^f_{\Sigma }(x,s) = 1$$.
  If $$f(x,s) \le f(x,s+1)$$, then $$m^f_{\Sigma }(x,s+1) = 1$$.
  If $$f(x,s) > f(x,s+1)$$, then $$m^f_{\Sigma }(x,s+1) = -1$$.
3. Suppose that $$m^f_{\Sigma }(x,s) = -1$$.
  If $$f(x,s) \ge f(x,s+1)$$, then $$m^f_{\Sigma }(x,s+1) = -1$$.
  If $$f(x,s) < f(x,s+1)$$, then $$m^f_{\Sigma }(x,s+1) = 1$$.

The $\Pi$-*mind-change function* $m^{f}_{\Pi }(x,s)$ is defined similarly to $m^f_{\Sigma }$, with the following key modification: we put $m^f_{\Pi }(x,0) = -1$.

Notice the following: if a function *f* is computable, then both $m_{\Sigma }^f$ and $m_{\Pi }^f$ are also computable. Now we are ready to give the main definition.

### Definition 5

Let *n* be a non-zero natural number. A fuzzy set *A* is *n*-*computably enumerable* if there is a computable function $f:\omega \times \omega \rightarrow [0,1]_{\mathbb {Q}}$ such that for all $x\in \omega$, we have:

- $$\lim _{s\rightarrow \infty } f(x,s)=A(x)$$;
- $$f(x,0) = 0$$;
- $$\vert \{ s\in \omega \,:m^f_{\Sigma }(x,s+1) \ne m^f_{\Sigma }(x,s)\}\vert \le n-1$$.

A fuzzy set *A* is *co*-*n*-*computably enumerable* if its complement $\overline{A}$ is *n*-c.e.

### Remark 2

Notice that the third condition of Definition 5 contains a modification—the upper bound *n* is changed to $n-1$ (cf. the third condition of Definition 3). This modification is of a technical nature. We illustrate the reason behind the modification by an example.

Let *A* be a crisp 3-c.e. set, and let *x* be a natural number. For simplicity, we assume that for this *x*, the function *f* from Definition 3 behaves as follows: $f(x,0) = 0$, $f(x,1) = 1$, $f(x,2) = 0$, and $f(x,s) = 1$ for all $s\ge 3$. So, the upper bound $n=3$ is achieved by the number *x*:

$$\begin{aligned} \left| \left\{ s \,:f(x,s) \ne f(x,s+1)\right\} \right| = 3 = n. \end{aligned}$$

Now we calculate the corresponding $\Sigma$-mind-change function: $m^f_{\Sigma }(x,0) = m^f_{\Sigma }(x,1) = 1$, $m^f_{\Sigma }(x,2) = -1$, and $m^f_{\Sigma }(x,s) = 1$ for all $s\ge 3$. We have

$$\begin{aligned} \vert \{ s \,:m^f_{\Sigma }(x,s+1) \ne m^f_{\Sigma }(x,s)\}\vert = 2 = n-1. \end{aligned}$$

The example illustrates the following: if $f(x,\cdot )$ changes its values at most *n* times, then the corresponding function $m^f_{\Sigma }(x,\cdot )$ can change its values only at most $n-1$ times. Since we want any crisp *n*-c.e. set to be also a fuzzy *n*-c.e. set, we have introduced the discussed modification in Definition 5.

Note that 1-c.e. fuzzy sets are precisely the c.e. sets from Definition 1. In addition, the following fact is immediate.

### Proposition 4

Let $n\ge 1$. A fuzzy set *A* is co-*n*-c.e. if and only if there is a computable function $f:\omega \times \omega \rightarrow [0,1]_{\mathbb {Q}}$ such that for all $x\in \omega$, we have:

- $$\lim _{s\rightarrow \infty } f(x,s)=A(x)$$;
- $$f(x,0) = 1$$;
- $$\vert \{ s\in \omega \,:m^f_{\Pi }(x,s+1) \ne m^f_{\Pi }(x,s)\}\vert \le n-1$$.

### The hierarchy does not collapse

In order to show the non-collapse of the hierarchy, it is sufficient to prove the following:

#### Proposition 5

Let *A* be a crisp subset of $\omega$. Then *A* is *n*-c.e. in the Classical Ershov Hierarchy if and only if *A* is *n*-c.e. in **FEH**. A similar fact is true for co-*n*-c.e. sets.

Indeed, since the Classical Difference Hierarchy does not collapse, Proposition 5 implies that our hierarchy is also non-collapsing.

#### Proof of Proposition 5

$(\Rightarrow )$. Suppose that *A* is *n*-c.e. in the classical sense. We fix a computable function $f:\omega ^2 \rightarrow \{ 0,1\}$ satisfying the conditions from Definition 3. It is clear that *f*(*x*, *s*) is a $\Delta ^0_2$-approximation of *A*, treated as a $\Delta ^0_2$ fuzzy set.

For an element $x\in \omega$, consider all stages $s_1< s_2<\dots < s_k$ (note that $k\le n$) such that $f(x,s_i) \ne f(x,s_i+1)$. A straightforward analysis shows the following:

- If $s \le s_1$, then $f(x,s) = 0$ and $m^{f}_{\Sigma }(x,s) = 1$.
- If $s_{2\ell +1} +1 \le s \le s_{2\ell +2}$, then $f(x,s) = 1$ and $m^{f}_{\Sigma }(x,s) = 1$.
- If $s_{2\ell +2} +1 \le s \le s_{2\ell +3}$, then $f(x,s) = 0$ and $m^{f}_{\Sigma }(x,s) = -1$.

This implies that $\vert \{ s\,:m^f_{\Sigma }(x,s+1) \ne m^f_{\Sigma }(x,s)\}\vert \le k-1 \le n-1$. We deduce that the approximation *f* witnesses that the fuzzy set *A* is *n*-c.e.

$(\Leftarrow )$. Suppose that a crisp *A* is *n*-c.e. Fix a $\Delta ^0_2$-approximation $f:\omega ^2 \rightarrow [0,1]_{\mathbb {Q}}$ satisfying Definition 5. We define a new approximation

$$\begin{aligned} g(x,s) = {\left\{ \begin{array}{ll} 1, &{} \text {if } f(x,s) > 1/2,\\ 0, &{} \text {if } f(x,s) \le 1/2. \end{array}\right. } \end{aligned}$$

Since *A* is crisp, it is clear that $A(x) = \lim _{s\rightarrow \infty } g(x,s)$. Notice that $g(x,0) = f(x,0) = 0$.

For an element $x\in \omega$, consider all stages $s_1< s_2< \dots < s_k$ such that $g(x,s_i) \ne g(x,s_i+1)$. For $i\le k$, one can show the following:

- If $i=2\ell +1$, then $f(x,s_i) \le 1/2$, $f(x,s_i +1) > 1/2$, and $m^{f}_{\Sigma }(x,s_i + 1) = 1$.
- If $i=2\ell +2$, then $f(x,s_i) > 1/2$, $f(x,s_i +1) \le 1/2$, and $m^{f}_{\Sigma }(x,s_i + 1) = -1$.

In turn, this implies $k-1 \le \vert \{ s\,:m^f_{\Sigma }(x,s+1) \ne m^f_{\Sigma }(x,s)\}\vert \le n-1$. Hence, $k\le n$, and the function *g*(*x*, *s*) witnesses that the set *A* is *n*-c.e. in the classical sense.

### Boolean combinations of fuzzy c.e. sets

We show that similarly to the Classical Ershov Hierarchy (Theorem 3), *n*-c.e. fuzzy sets admit natural presentations via Boolean combinations of c.e. sets.

#### Theorem 6

Let $n\in \{2 k+1, 2k+2\}$. A fuzzy set *C* is *n*-c.e. if and only if there are c.e. fuzzy sets $A_1,B_1,A_2,B_2,\dots ,A_{k+1},B_{k+1}$ such that:

- $$C = (A_1 \cap \overline{B_1}) \cup (A_2 \cap \overline{B_2}) \cup \dots \cup (A_{k+1} \cap \overline{B_{k+1}})$$;
- if $$n = 2k+1$$, then $$B_{k+1} = \emptyset $$.

#### Proof

$(\Rightarrow )$. Let *f*(*x*, *s*) be a $\Delta ^0_2$-approximation which witnesses the fact that *C* is *n*-c.e. We define the desired c.e. fuzzy sets $A_i$ and $B_i$ via their $\Sigma ^0_1$-approximations $h_{A_i}$ and $h_{B_i}$ (in the sense of Definition 1), respectively.

The intuition behind these c.e. sets is as follows. For an element $$x\in \omega $$, we split $$\omega $$ into disjoint intervals: $$[0,a_0)$$, $$[a_0,b_0)$$, $$[b_0,a_1)$$, $$[a_1,b_1)$$, etc. Our function $$f(x,\cdot )$$ (non-strictly) increases on the intervals $$[0,a_0)$$, $$[b_0,a_1)$$, $$[b_1,a_2)$$, etc. The function decreases on the rest of the intervals.

- The approximation $$h_{A_1}$$ of the set $$A_1$$ looks like this: it copies $$f(x,\cdot )$$ on the interval $$[0,a_0)$$, and then stabilizes, i.e. $$h_{A_1}(x,s) = h_{A_1}(x,a_0-1)$$ for all $$s\ge a_0$$.
- The function $$h_{B_1}(x,\cdot )$$ equals zero on $$[0,a_0)$$. Then it copies $$1-f(x,\cdot )$$ on the interval $$[a_0,b_0)$$. After that, $$h_{B_1}(x,\cdot )$$ equals *one*.
- The function $$h_{A_2}$$ equals zero on $$[0,b_0)$$. Then it copies $$f(x,\cdot )$$ on the interval $$[b_0,a_1)$$; after that $$h_{A_2}$$ stabilizes.
- Et cetera.

Formally speaking, for a non-zero $i \le k+1$, we define:

$$\begin{aligned} h_{A_i}(x,s)= & {} {\left\{ \begin{array}{ll} 0, &{} \text {if } \vert \{ t\le s \,:m^{f}_{\Sigma }(x,t+1) \ne m^{f}_{\Sigma }(x,t)\}\vert< 2i-2,\\ f(x,s), &{} \text {if } \vert \{ t\le s \,:m^{f}_{\Sigma }(x,t+1) \ne m^{f}_{\Sigma }(x,t)\}\vert = 2i-2,\\ h_{A_i}(x,s-1), &{} \text {otherwise}; \end{array}\right. }\\ h_{B_i}(x,s)= & {} {\left\{ \begin{array}{ll} 0, &{} \text {if } \vert \{ t\le s \,:m^{f}_{\Sigma }(x,t+1) \ne m^{f}_{\Sigma }(x,t)\}\vert < 2i-1,\\ 1-f(x,s), &{} \text {if } \vert \{ t\le s \,:m^{f}_{\Sigma }(x,t+1) \ne m^{f}_{\Sigma }(x,t)\}\vert = 2i-1,\\ 1, &{} \text {otherwise}. \end{array}\right. } \end{aligned}$$

It is not hard to see that these approximations induce c.e. fuzzy sets. In addition, if $n=2k+1$, then $B_{k+1}(x) = 0$ for all *x*.

Let *D* be the fuzzy set $(A_1 \cap \overline{B_1}) \cup \dots \cup (A_{k+1} \cap \overline{B_{k+1}})$. We consider its natural $\Delta ^0_2$-approximation

1 $$\begin{aligned} h_D(x,s) = \max \{ \min \{ h_{A_i}(x,s), 1-h_{B_i}(x,s)\} \,:1\le i \le k+1\}. \end{aligned}$$

Consider the value $v^{*} = \vert \{ t\in \omega \,:m^{f}_{\Sigma }(x,t+1) \ne m^{f}_{\Sigma }(x,t)\}\vert$.

If $v^{*} = 2i-2$, then there is a stage $s^{*}$ such that for all $s\ge s^{*}$, we have $h_D(x,s) = h_{A_i}(x,s) = f(x,s)$. This implies that $D(x) = C(x)$.

If $v^{*} = 2i-1$, then consider the highest index $s_0$ such that $h_{A_i}(x,s_0) = f(x,s_0)$. Then for every $s\ge s_0 +1$, we have $h_{A_i}(x,s) = f(x,s_0)$, $h_{B_i}(x,s) = 1 - f(x,s)$, and

$$\begin{aligned} h_{D}(x,s) = \min (h_{A_i}(x,s), 1-h_{B_i}(x,s)) = \min (f(x,s_0), f(x,s)) = f(x,s). \end{aligned}$$

Again, $D(x) = C(x)$. We deduce that the fuzzy sets *C* and *D* are equal.

$(\Leftarrow )$. Let *D* be a $\Delta ^0_2$ fuzzy set defined via the approximation $h_D$ from (1). We prove that *this* approximation $h_D$ witnesses the fact that *D* is *n*-c.e.

First, we note the following easy observation (it follows from computable enumerability of fuzzy sets $A_i$ and $B_i$):

($$*$$)
If $1-h_{B_i}(x,s_0) < h_{A_i}(x,s_0)$ for some $s_0$, then we have $1-h_{B_i}(x,s) < h_{A_i}(x,s)$ *for all* $s\ge s_0$.

An informal intuition concerning further proof is as follows. Every (approximation of the) real $(A_i \cap \overline{B}_i)(x)$ can be treated as a “hill”: first we go up, copying the function $h_{A_i}(x,\cdot )$. When we see the inequality $1-h_{B_i}(x,s_0) < h_{A_i}(x,s_0)$, we can only go down. Coming back to the whole picture of $h_D$: whenever the mind-change function $m^{h_D}_{\Sigma }(x,\cdot )$ changes from $+1$ to $-1$, it happens because we encountered the *top* of one of the “hills”.

At a stage *s*, consider the following sets: $X_s = \{ i\,:1-h_{B_i}(x,s) < h_{A_i}(x,s)\}$ and $Y_s = \{1,2,\dots ,k+1\} {\setminus } X_s$. Observation $(*)$ implies that $X_{s} \subseteq X_{s+1}$ for every *s*. In addition, $X_0=\emptyset$.

It is not hard to deduce the following equation:

$$\begin{aligned} h_D(x,s) = \max \{ \max \{ 1-h_{B_i}(x,s)\,:i \in X_s\}, \max \{ h_{A_i}(x,s)\,:i \in Y_s\} \}. \end{aligned}$$

Note that for a fixed non-empty set *Z*, the function $\max \{ 1-h_{B_i}(x,s)\,:i \in Z\}$ is non-increasing, and $\max \{ h_{A_i}(x,s)\,:i \in Z\}$ is non-decreasing.

Suppose that $m^{h_D}_{\Sigma }(x,s) = 1$ and $m^{h_D}_{\Sigma }(x,s+1) = -1$. Choose the greatest $s'< s$ such that either $s'=0$, or $s'>0$ and $m^{h_D}_{\Sigma }(x,s')=-1$. Towards a contradiction, assume that $X_{s+1}=X_{s'}$.

Then on one hand, we have

$$\begin{aligned} h_D(x,s) = \max \{ 1-h_{B_i}(x,s)\,:i \in X_{s'}\} > \max \{ h_{A_i}(x,s)\,:i \in Y_{s'}\}. \end{aligned}$$

Indeed, if $h_D(x,s)$ equals $\max \{ h_{A_i}(x,s)\,:i \in Y_{s'}\}$, then we would have

$$\begin{aligned} h_D(x,s+1) = \max \{ h_{A_i}(x,s+1) \,:i \in Y_{s'}\} \ge h_D(x,s), \end{aligned}$$

which contradicts the fact that $m^{h_D}_{\Sigma }(x,s+1) = -1$.

On the other hand, every *t* such that $s'< t \le s$ satisfies

$$\begin{aligned} h_D(x,t) = \max \{ h_{A_i}(x,t)\,:i \in Y_{s'} \}. \end{aligned}$$

We obtain a contradiction. Therefore, $X_{s+1}\ne X_{s'}$.

We deduce that for each stage *s* with $m^{h_D}_{\Sigma }(x,s) = 1$ and $m^{h_D}_{\Sigma }(x,s+1) = -1$, at least one new element is added to the growing set $X = \bigcup _{t\in \omega } X_t$.

Suppose that $n=2k+2$. Then one can show that $\vert X \vert \le k+1$. We notice the following: if $\vert X \vert$ is less than $k+1$, then the number of monotonicity breaks (of the function $m^{h_D}_{\Sigma }(x,\cdot )$) will be strictly less than the corresponding number for the case $\vert X \vert =k+1$. Hence, one can consider only the case when $\vert X \vert =k+1$.

If $\vert X \vert =k+1$, then there is a stage $s^{*}$ such that for all $s\ge s^{*}$, we have $h_D(x,s) = \max \{ 1-h_{B_i}(x,s)\,:i \in X\}$, and this function can only decrease. A not difficult combinatorial argument shows that $\vert \{ s\in \omega \,:m^{h_D}_{\Sigma }(x,s+1) \ne m^{h_D}_{\Sigma }(x,s)\}\vert \le 2k+1$.

If $n=2k+1$, then $\vert X\vert \le k$. An argument similar to the one above shows that one can consider only the case when $\vert X\vert$ equals *k*.

If $\vert X\vert =k$, then there is a stage $s^{*}$ such that for $s\ge s^{*}$, we have

$$\begin{aligned} h_D(x,s) = \max \{ \max \{ 1-h_{B_i}(x,s)\,:i \in X \}, h_{A_{k+1}}(x,s)\}. \end{aligned}$$

One can show that in this case, $\vert \{ s\,:m^{h_D}_{\Sigma }(x,s+1) \ne m^{h_D}_{\Sigma }(x,s)\}\vert \le 2k$. Theorem 6 is proved.

#### Corollary 7

Every finite Boolean combination of c.e. fuzzy sets is an *n*-c.e. set, for some $n\ge 1$.

### The introduced hierarchy is not enough

Here we show that the introduced levels of **FEH** do not exhaust the class of all $\Delta ^0_2$ fuzzy subsets of $\omega$.

#### Proposition 8

There exists a $\Delta ^0_2$ fuzzy set *A* such that for any $\Delta ^0_2$-approximation *f*(*x*, *s*) of *A*, the sequence $(m^f_{\Sigma }(0,s))_{s\in \omega }$ diverges when *s* tends to infinity. In particular, *A* is not *n*-c.e., for all $n\ge 1$.

#### Proof

Choose an arbitrary $\Delta ^0_2$ real $\alpha$, which is not left-c.e. and not right-c.e. (see, e.g., Theorem 5.1.10 in Downey and Hirschfeldt (2010) for an example of such real). The desired fuzzy set *A* is defined as follows: put $A(k) = \alpha$, for all $k\in \omega$. Since $\alpha$ is $\Delta ^0_2$, Proposition 2 implies that the set *A* is $\Delta ^0_2$.

Towards a contradiction, assume that *f*(*x*, *s*) is a $\Delta ^0_2$-approximation of *A* such that the sequence $(m^f_{\Sigma }(0,s))_{s\in \omega }$ converges. There are two possible cases.

*Case 1* Suppose that $\lim _{s\rightarrow \infty } m^f_{\Sigma }(0,s) = 1$. Then choose a stage $s^{*}$ such that $m^f_{\Sigma }(0,s) = 1$ for all $s\ge s^{*}$. It is not hard to show that the set $\{ q\in \mathbb {Q} \,:q < \alpha \}$ is equal to $\{ q\,:(\exists s \ge s^{*})[ q < f(0,s) ]\}$, and hence, this set is c.e. Then the real $\alpha$ is left-c.e., which gives a contradiction.

*Case 2* Otherwise, $\lim _{s\rightarrow \infty } m^f_{\Sigma }(0,s) = -1$. Then a similar argument shows that the real $\alpha$ is right-c.e.—again, a contradiction.

We deduce that our fuzzy set *A* has all desired properties.

## Broadening the Fuzzy Ershov Hierarchy

In this section, we investigate two natural options for, first, *refining* the $\textbf{FEH}$, and, secondly, *extending* the finite levels of the hierarchy.

### Counting updates

We say that a $\Delta ^0_2$-approximation *f* of a fuzzy set *A* *has an update* if $f(x,s+1)\ne f(x,s)$, for some $x,s\in \omega$. Observe that our notion of mind-change, as in Definition 5, keeps track only of those updates which determine a change of monotonicity in the approximating function: e.g., if *f*(*x*, *s*) is a $\Delta ^0_2$-approximation of a fuzzy set *A*, $m^f_{\Sigma }(x,s)=1$, and $f(x,s+1)>f(x,s)$, then $m^f_{\Sigma }(x,s+1)$ remains equal to 1. So, one may explore what happens if one keeps track of *all* updates for a given $\Delta ^0_2$-approximation. To motivate such approach, consider the following example given by Harkleroad (1984).

#### Example 1

As usual, *K* denotes the Halting problem. Define

$$\begin{aligned} H(x) = {\left\{ \begin{array}{ll} 1 &{} \text {if } x \in K,\\ \frac{1}{2} &{} \text {otherwise}. \end{array}\right. } \end{aligned}$$

It is easy to see that *H* is a c.e. fuzzy set. But note that, for any c.e. approximation *h* of *H* (recall that one assumes $h(x,0) = 0$ for all *x*), there must be an infinite crisp set $Z\subseteq \omega$ such that *h* requires at least *two* updates to approximate each $x\in Z$, as otherwise, $K=\{x \,:(\exists s)(h(x,s)=1) \}$ would be computable.

So, to distinguish *H* from those c.e. fuzzy sets which can be approximated with at most one update, we propose the following definition.

#### Definition 6

A fuzzy set *A* is $[n]_1$-*c.e.* if there is a $\Delta ^0_2$ approximation *f*(*x*, *s*) to *A* such that, for all *x*:

- $$f(x,0) = 0$$;
- $$(\forall s)(f(x,s+1)\ge f(x,s))$$;
- $$\vert \{s\,:f(x,s+1)\ne f(x,s)\}\vert \le n$$.

It is immediate to note that, for all *n*, every $[n]_1$-c.e. is a c.e. fuzzy set. The next result establishes that the hierarchy of $[n]_1$-c.e. sets does not collapse, but it also doesn’t exhaust the class of all c.e. fuzzy sets.

#### Proposition 9

The following hold:

1. for all *n*, there is a $[n+1]_1$-c.e. set which is not $[n]_1$-c.e.;
2. there is a c.e. fuzzy set which is not $[n]_1$-c.e., for all *n*.

#### Proof

For the sake of exposition, we first re-prove that there is a $[2]_1$-c.e. set which is not $[1]_1$-c.e. (as is illustrated by Example 1). In doing so, we will suitably modify Harkleroad’s example, obtaining a module which is apt to be generalized.

Let *U* be a crisp 2-c.e. set which is not c.e., that is, $U\in \Sigma ^{-1}_2\smallsetminus \Sigma ^{-1}_1$. By Theorem 3, there exist c.e. sets $W_1, W_2$ so that $U=W_1\smallsetminus W_2$. Without loss of generality, one may assume that $W_2 \subseteq W_1$: indeed, if $W_2 \not \subseteq W_1$, then we replace $W_1$ with the c.e. set $W^{\text {new}}_1:= W_1 \cup W_2$. Then we define the following c.e. fuzzy set:

$$\begin{aligned} H_2(x):={\left\{ \begin{array}{ll} 1 &{}\text {if}\,x\in W_2,\\ \frac{1}{2} &{}\text {if}\,x\in W_1\hbox { and }x \notin W_2,\\ 0 &{}\text {otherwise}. \end{array}\right. } \end{aligned}$$

It is easy to see that $H_2$ is $[2]_1$-c.e. Indeed, it suffices to define a $\Delta ^0_2$-approximation $g_0$ which, on input *x*, has an update if, at some stage $s_0$, *x* is enumerated into $W_1$ but not into $W_2$, and then let $g_0$ have the second (and last) update if, at stage $s_1>s_0$, *x* is also enumerated into $W_2$.

On the other hand, suppose that there is a $\Delta ^0_2$-approximation $g_1$ which witnesses that $H_2$ is $[1]_1$-c.e. Since $g_1$ can have at most one update for each input, we would have that

$$\begin{aligned} W_1 \smallsetminus W_2 = \{ x: (\exists s)(f(x,s) = 1/2)\}, \end{aligned}$$

contradicting the fact that *U* is not c.e.

More generally, to construct a fuzzy set which is $[n+1]_1$-c.e. but not $[n]_1$-c.e., take a crisp set $V\in \Sigma ^{-1}_{n+1}\smallsetminus \Sigma ^{-1}_{n}$. Without loss of generality, assume that *n* is even. Theorem 3 guarantees that there are c.e. sets $W_1, W_2, \ldots , W_{n+1}$ so that

$$\begin{aligned} V=(W_1\smallsetminus W_2) \cup \cdots \cup (W_{n-1}\smallsetminus W_n) \cup W_{n+1}. \end{aligned}$$

We assume that

$$\begin{aligned} W_{n+1} \subseteq W_n \subseteq W_{n-1} \subseteq \dots \subseteq W_1. \end{aligned}$$

As usual, this condition can be achieved in a “dynamic” way: For a non-zero $i\le n+1$, the new set $W^{\text {new}}_{i}$ includes all numbers *x* such that there exists a sequence of stages $0 = s_0< s_1< s_2< \dots < s_i$ with the following properties:

- for odd $j\le i$, the element *x* belongs to the finite set
  $$\begin{aligned} V_{s_j} = (W_{1,s_j} \smallsetminus W_{2,s_j}) \cup \cdots \cup (W_{n-1,s_j}\smallsetminus W_{n,s_j}) \cup W_{n+1,s_j}, \end{aligned}$$
  where for $s\in \omega$, the finite set $W_{k, s}$ contains all elements of $W_k$ enumerated by the stage *s*;
- for even $j\le i$, the number *x* does not belong to the set $V_{s_j}$.

Next, define $H_{n+1}$ as follows,

$$\begin{aligned} H_{n+1}(x):={\left\{ \begin{array}{ll} 1 &{}\text {if}\,x\in W_{n+1},\\ \frac{1}{2} &{}\text {if}\,x\in W_n\hbox { and }x \notin W_{n+1},\\ \ldots \\ \frac{1}{n-k+2} &{}\text {if}\,x\in W_k\hbox { and }x \notin W_{k+1},\\ \ldots \\ \frac{1}{n} &{}\text {if}\,x\in W_2\hbox { and }x \notin W_3,\\ \frac{1}{n+1} &{}\text {if}\,x\in W_1\,\hbox {and } x \notin W_2,\\ 0 &{}\text {otherwise}. \end{array}\right. } \end{aligned}$$

The fuzzy set $H_{n+1}$ is clearly $[n+1]_1$-c.e.. By reasoning as above, it is not hard to deduce that it cannot be $[n]_1$-c.e. (or, a fortiori, $[k]_1$-c.e. for any $k<n$). Indeed, suppose that there is a $\Delta ^0_2$-approximation $g_2$ witnessing that $H_{n+1}$ is $[n]_1$-c.e.. Then, we would be able to approximate whether any given *x* belongs to *V* with at most *n* mind-changes, contradicting the choice of such *V*.

(2) To construct a c.e. fuzzy set which is not $[n]_1$-c.e., for all *n*, it suffices to *join* all the sets $H_n$’s defined in item (1). For all *x* and *n*, let

$$\begin{aligned} \widehat{H}(\langle x,n\rangle ):= H_n(x), \end{aligned}$$

where $\langle \cdot , \cdot \rangle$ denotes a Cantor pairing function, i.e., an effective bijection from $\omega ^2$ onto $\omega$. Towards a contradiction, suppose that there is a $\Delta ^0_2$-approximation $h_0$ witnessing that $\widehat{H}$ is $[n]_1$-c.e., for some *n*. Let $m>n$ and define

$$\begin{aligned} h_1(x,s):=h_0(\langle x,m\rangle , s), \end{aligned}$$

for all *x* and *s*. Then, $h_1$ would witness that $H_m$ is $[n]_1$-c.e., a contradiction.

Now, we shall similarly stratify each level of **FEH**, by counting the number of updates that are needed to approximate *n*-c.e. fuzzy sets. Intuitively, we say that a fuzzy set *A* is $[n_1,\ldots ,n_m]_m$-c.e., if there is a $\Delta ^0_2$-approximation *f* of *A* that, for each input *x*, can go up at most $n_1$ times, and then go down at most $n_2$ times, etc.—for *m*-many ups and downs.

More formally, let *f* be a $\Delta ^0_2$-approximation of a fuzzy set *A*. For simplicity, we shall also assume that, for all *x*, $f(x,0)=0$. Let $\alpha \in \omega ^\omega$ and denote $\alpha$ as $(\alpha _0,\alpha _1,\ldots ,\alpha _k,\ldots )$. To calculate if $\alpha$ bounds the number of updates produced by *f*, we shall run the following algorithm (called Bounding) for all $x\in \omega$:

- *Stage* 0. Set $\gamma$ to 0 and let $\nu (x,0):=\alpha _\gamma$.
- *Stage* $s+1$. We distinguish two cases:
  (i) $m^f_\Sigma (x,s+1)= m^f_\Sigma (x,s)$ (that is, *f* preserves its current monotonicity on *x*). Then,
    $$\begin{aligned} \nu (x,s+1):={\left\{ \begin{array}{ll} \nu (x,s)-1 &{}\text {if } f(x,s+1)\ne f(x,s),\\ \nu (x,s) &{}\text {otherwise}. \end{array}\right. } \end{aligned}$$
  (ii) $m^f_\Sigma (x,s+1)\ne m^f_\Sigma (x,s)$ (that is, *f* changes its current monotonicity on *x*). Then update the value $\gamma$ to $\gamma +1$. Next, let $\nu (x,s+1):=\alpha _\gamma -1$.
  The algorithm returns $\textbf{no}$, if $\nu (x,s+1)<0$.

If Bounding never outputs $\textbf{no}$, for all input *x*, then we say that *f* is *bounded* by $\alpha$.

#### Definition 7

Let $(n_0,n_1,\ldots ,n_k)$ be a tuple of natural numbers containing no zeros. A fuzzy set *A* is $[n_0,n_1,\ldots ,n_k]_{k}$-c.e. if there is a $\Delta ^0_2$-approximation *f* of *A* which is bounded by

$$\begin{aligned} {n_0}^{\frown }{n_1}^\frown \cdots ^\frown n_k^\frown {0^\infty }, \end{aligned}$$

where $0^\infty$ denotes the sequence consisting of infinitely many zeros.

#### Remark 3

Let $\rho \in \omega ^k$ be a *k*-tuple of natural numbers. For notational simplicity, if a $\Delta ^0_2$-approximation *f* is bounded by $\rho ^\frown 0^\infty$, we may just say that *f* is $\rho$-bounded. Similarly, we may simply write that *A* is $\rho$-c.e. (rather than $[\rho ]_k$-c.e.).

By the last definition, we have refined **FEH** with a plethora of new sub-levels. It is natural to ask how these sub-levels compare with each other. For example, can there be a fuzzy set which is $[2,1,1]_3$-c.e. but not $[1,10,10]_3$-c.e.? At first sight, one may guess that the answer is no, as it may seem plausible that the behavior of a $\Delta ^0_2$-approximation *f* which is constrained to have at most 4 updates, for each input, could be always emulated by a function bounded by (1, 10, 10). But this is not the case.

#### Theorem 10

For every natural number *k*, the class of $[n_0,n_1,\ldots ,n_k]_k$-c.e. sets is contained in the class of $[m_0,m_1,\ldots ,m_k]_k$-c.e. sets if and only if

$$\begin{aligned} (n_0,n_1,\ldots ,n_k) \text{ is } \text{ bitwise } \text{ below } (m_0,m_1,\ldots ,m_k), \end{aligned}$$

that is, $n_i\le m_i$, for all $i\le k$.

#### Proof

Let $\rho$, $\sigma$ be *k*-tuples with $\rho$ bitwise below $\sigma$. It follows immediately from Definition 7 that, if *f* is $\rho$-bounded, then *f* is also $\sigma$-bounded. Hence, any fuzzy set which is $\rho$-c.e. must also be $\sigma$-c.e.

On the other hand, suppose that $\rho =(n_0,\ldots ,n_k)$ is not bitwise below $\sigma =(m_0,\ldots ,m_k)$. In particular, let *i* be the least number so that $n_i>m_i$. For the sake of exposition, assume that *i* is even (the other case being symmetric): this means that $n_i$ correspond to a sequence of *increasing* updates. Now, we shall construct a fuzzy set *A* which is $\rho$-c.e. but not $\sigma$-c.e. To do so, we will construct by stages a $\Delta ^0_2$-approximation *f* of *A* which meet the following infinite list of *requirements*:

- $$\mathcal {P}$$: *f* is $$\rho $$-bounded;
- $\mathcal {R}_e$: If the function $\phi _e$ is $\sigma$-bounded, then
  $$\begin{aligned} \lim _{s\rightarrow \infty }(f(e,s))\ne \lim _{s\rightarrow \infty }(\phi _e(e,s)). \end{aligned}$$

Note that the combination of all $\mathcal {R}$-requirements ensures that *A* cannot be approximated by any function which is $\sigma$-bounded.

Through this construction, it will be very convenient to view *f* dynamically. That is, rather than defining *f*(*x*, *s*), for all *s*, we just let *f*(*x*) change its value during the construction, and we will interpret $\lim _{s\rightarrow \infty }(f(x,s))$ as the limit value (if any) of such a sequence of changes. Similarly, by referring to the *current value* of $\phi _e(e)$, we simply mean $\phi _e(e,s)$, for the current stage *s* of the construction.

**Strategy to meet the requirements** For a computable function $\phi _e$, the property of being $\sigma$-bounded is $\Pi ^0_1$. But to address $\mathcal {R}_e$, it suffices to restrict the focus to the behavior of the algorithm Bounding on *e*, up to *k* many changes of monotonicity.

The basic idea of the strategy is straightforward. We shall act whenever we witness that the current value of *f*(*e*) coincides with the current value of $\phi _e(e)$. Yet, our actions are implemented in different ways, corresponding to three distinct phases:

- In Phase I, to ensure that *f*(*e*) differs from $\phi _e(e)$ we simply alternate, whenever is necessary, between 0 and 1. That is, if we witness that
  $$\begin{aligned} f(e)=\phi _e(e)=j, \end{aligned}$$
  for $j\in \{0,1\}$, we respond by updating *f*(*e*) to $1-j$. We perform this action for at most *i* times. Then we move to Phase II.
- When we enter Phase II, both *f* and $\phi _e$ have changed monotonicity (on input *e*) $i-1$ times. Moreover, since we are under the assumption that *i* is even, we have that the value of *f*(*e*), when Phase II starts, is 0. Now, $\rho$ bounds *f* to a sequence of $n_i$ many increasing updates, while $\sigma$ bounds $\phi _e$ to only $m_i$ many increasing updates. We take advantage of the fact that $n_i>m_i$ by performing two sorts of actions:
  (i) If we witness that $f(e)=\phi _e(e)$, we respond by increasing the current value of *f*(*e*) to the midpoint between *f*(*e*) and 1, that is, we update *f*(*e*) to $\frac{f(e)+1}{2}$. If we also see that $\phi _e$ exhausted all its $m_i$ many updates, we move to the Transition phase.
  (ii) If we witness that $f(e)<\phi _e(e)$, we immediately move to the Transition phase.
- We may start the Transition phase with either $f(e)>\phi _e(e)$ or $f(e)<\phi _e(e)$:
  - In the first case, we wait to see if, at some further stage, $\phi _e(e)$ equals $f_e(e)$. If this happens, we let $f(e)=0$ and we go back to Phase I.
  - In the second case, we again wait to see if $\phi _e(e)$ equals $f_e(e)$. If this happens, we now let $f(e)=1$ and we go back to Phase I.

There are no interactions between different strategies.

**The construction** At any stage, each requirement, independently from all others, can either be in Phase I, or in Phase II, or in the Transition phase. We use a collection of dynamic counters $(c_e)_{e\in \omega }$ to record how many times we have changed the monotonicity of *f*. Recall that $\rho$ is of the form $(n_0,n_1,\ldots ,n_k)$, hence in defining *f* we are constrained to at most *k* many changes of monotonicity. We also use a collection of parameters $(d_e)_{e\in \omega }$, which range on the set $\{-1,1\}$, to distinguish the actions of the Transition phase. Finally, at any given stage *s*, $\phi _e$ is $\sigma$-*compatible*, if the algorithm Bounding on input *e* does not return $\textbf{no}$ within *s* many stages.

- *Stage* 0. For all *e*, let $c_e=d_e=0$. Say that every requirement $\mathcal {R}_e$ is in Phase I.
- *Stage* $s+1=\langle e, t\rangle$. We deal with $\mathcal {R}_e$. If $\phi _e$ is not $\sigma$-compatible or $c_e>k$, then we do nothing. Otherwise, we act accordingly to the current phase of $\mathcal {R}_e$.
  - $\mathcal {R}_e$ is in Phase I and the current value of $f_e(e)$ is *j*, for $j\in \{0,1\}$: If we witness that $\phi _e(e)$ currently equals *j*, we update the value of $f_e(e)$ to $1-j$. We then increase the counter $c_e$ by 1. If, after this action, $c_e$ equals *i*, we enter Phase II; otherwise, we remain in Phase I.
  - $\mathcal {R}_e$ is in Phase II and the current value of $f_e(e)$ is *u*, for $u\in [0,1)$: We distinguish three sub-cases, corresponding to the current value of $\phi _e(e)$:
    1. If $\phi _e(e)<u$, we do nothing and we remain in Phase II;
    2. If $\phi _e(e)=u$, we update *f*(*e*) to $\frac{f(e)+1}{2}$. If it is also the case that $\phi _e(e)$ already had $m_i$ many consecutive increasing updates, we set $d_e$ to 1 and we move to the Transition phase; otherwise, we remain in Phase II;
    3. If $\phi _e(e)>u$, we set $d_e$ to $-1$ and then we move to the Transition phase.
  - $\mathcal {R}_e$ is in the Transition phase and the current value of $f_e(e)$ is *v*, for $v\in [0,1]$: If we witness that $\phi _e(e)$ currently equals *v*, we distinguish two sub-cases:
    1. If $d_e=1$, we update the value of *f*(*e*) to 0. Then, we increase the counter $c_e$ by 1, and we go back to Phase I;
    2. If $d_e=-1$, we update the value of *f*(*e*) to 1 and we go back to Phase I, without updating $c_e$.

This concludes the construction.

**The verification** To conclude that *A* is $\rho$-c.e. but not $\sigma$-c.e., it is sufficient to show that all requirements are eventually satisfied. First, note that the construction ensures that the evolution of phases of each $\mathcal {R}$-requirement follows the next diagram (possibly completing only an initial segment of it):

$$\begin{aligned} \texttt {Phase I}\rightarrow \texttt {Phase II}\rightarrow \texttt {Transition phase} \rightarrow \texttt {Phase I}. \end{aligned}$$

To see that the global $\mathcal {P}$-requirement is satisfied, it is sufficient to note that, for all *e*, the following hold:

1. In Phase I, each action forces *f* to change monotonicity on *e*. The same is true for the Transition phase with $d_e=1$. So, for all $i^* \ne i$ with $i^*\le k$, we have that *f* consumes only 1 update and so it is certainly bounded by $n_{i^*}$;
2. The updates consumed by *f* in Phase II must be bounded by $n_i$. This is satisfied, as the construction ensures that, in this phase, *f* has:
  at most $m_i+1$ many updates (with $m_i<n_i$), if $d_e = 1$; or
  at most $m_i$ many updates, plus one additional update in the subsequent Transition phase if $d_e=-1$.
3. Finally, if *f* changes monotonicity *k* many times, then no further action is allowed, and thus *f* has no more updates on *e*.

Thus, *f* is $\rho$-bounded. But, in fact, it is even bounded by $(1,\ldots ,1,n_i,1,\ldots ,1)$.

It remains to prove that all $\mathcal {R}$-requirements are satisfied. Suppose otherwise. Let *e* be so that $\phi _e$ is $\sigma$-bounded and, for all *x*, the limit value of $\phi _e(x)$ coincides with the limit value of $f_e(x)$. Let *s* be the first stage such that, after *s*, neither *f*(*e*) nor $\phi _e(e)$ are updated. Towards a contradiction, we now discuss in which phase $\mathcal {R}_e$ is, at the least stage $\langle e, t\rangle >s$. Suppose that, at this stage, $\mathcal {R}_e$ is in Phase I with $c_e<i$. If so, the construction allows to update *f*(*e*) to either 0 or 1, ensuring that *f*(*e*) differs from $\phi _e(e)$, a contradiction. Next, suppose that $\mathcal {R}_e$ is in Phase II. If so, since $n_i>m_i$, the construction allows again to make *f*(*e*) different from $\phi _e(e)$, a contradiction. A similar reasoning excludes that $\mathcal {R}_e$ is in the Transition phase.

The only remaining case is that $\mathcal {R}_e$ is in Phase I with $c_e \ge i$. Now, the key observation is that, when $\mathcal {R}_e$ visits $\texttt {Phase I}$ for the second time, it must be the case that *f*(*e*) consumed strictly less changes of monotonicity than $\phi _e(e)$. To see this, we reconstruct the behavior of *f* and $\phi _e$ on *e*, when moving from Phase II through the Transition phase to Phase I. When Phase II ends, both *f* and $\phi$ had *i* many changes of monotonicity on input *x*. Then we separate two cases. If $d_e=-1$, then $\phi _e(e)$ needs to change monotonicity to reach *f*(*e*), but we respond by increasing *f*(*e*) to 1 without changing the monotonicity of *f* (see item 2 of the Transition phase above). Hence, if $\mathcal {R}_e$ is in Phase I at stage $\langle e,t\rangle$, then, from the fact that $\phi _e(e)$ is again equal to *f*(*e*), we deduce that, on input *e*, $\phi _e$ and *f* changed monotonicity $i+2$ and $i+1$ many times, respectively. The case $d_e=1$ is similar. Note in particular that, in this case, Phase II ends with $f(e)>\phi _e(e)$ and the next sequence of updates that $\phi _e$ can use are decreasing. Hence, it requires two changes of monotonicity for $\phi _e(e)$ to reach *f*(*e*) (this is vacuously true, even if $\phi _e$ consumes no decreasing update from the $n_{i+1}$ sequence, as $\phi _e$ would still need to consume the $n_{i+2}$ sequence to copy *f*). It follows that, if $d_e=1$, $c_e\ge i$, $\mathcal {R}_e$ is in Phase I, and $\phi _e(e)=f(e)$, then, on input *e*, $\phi _e$ and *f* changed monotonicity $i+3$ and $i+1$ many times, respectively.

To sum up, if $\phi _e$ copies the behavior of *f* enough times, then the construction reaches a stage in which $\mathcal {R}_e$ is in Phase I and *f*(*e*) has consumed strictly less changes of monotonicity than $\phi _e(e)$. Such a difference in the number of changes of monotonicity of *f* and $\phi _e$ will then be preserved by any further action of Phase I. So, we will be able to respond to any threat of $\phi _e$, guaranteeing that eventually *f*(*e*) will differ from $\phi _e(e)$.

This proves that all $\mathcal {R}$-requirements are satisfied. Thus, *A*, which is $\rho$-bounded, cannot be approximated by a function which is $\sigma$-bounded. This concludes the proof of Theorem 10.

We just proved that the refined hierarchy discussed in this subsection is quite wild. It is now time to discuss how to *extend* $\textbf{FEH}$.

### Going transfinite

As is shown in Proposition 8, there are $\Delta ^0_2$ fuzzy sets which are not *n*-c.e., for all $n\ge 1$. Let us consider another example of such a set.

Fix an effective enumeration of all c.e. fuzzy sets $\{V_e\}_{e\in \omega }$; one can recover such an enumeration by applying Proposition 1 and re-arranging the enumeration of some left-c.e. reals (as in Herbert et al. (2019)). With the help of Theorem 6, we could also construct an effective uniform enumeration $\{V^n_e\}_{e\in \omega }$ of all *n*-c.e. fuzzy sets, for all $n\ge 1$. Since each $V^n_e$ is an *n*-c.e. set, there is a computable function $f^n(e,x,s)$ so that:

- $$\lim _{s\rightarrow \infty } f^n(e,x,s)=V^n_e(x)$$;
- $$f^n(e,x,0) = 0$$.

Next, we define a computable function *g* by diagonalization. That is, for all natural numbers *n*, *e*, *x*, let

- $$g(x,0)=0$$;
- $$g(\langle n,e,x\rangle ,s+1)=1-f^n(e,\langle n,e,x\rangle ,s)$$;

The fuzzy set *B*(*x*), defined as $\lim _{s\rightarrow \infty } g(x,s)$, is $\Delta ^0_2$, but not *n*-c.e. for all $n\ge 1$. Indeed, assume that *B* is *n*-c.e. Then, there must be *e* so that $B = V^n_e$. But this contradicts the fact that the definition of *g* ensures that

$$\begin{aligned} B(\langle n,e,x\rangle ) = 1-V^n_e(\langle n,e,x\rangle ), \end{aligned}$$

for every $$x\in \omega $$.

Note that the fuzzy set *B* just defined is significantly different from the set *A* constructed in Proposition 8. For the set *A*, the $\Sigma$-mind-change sequence $(m^{f}_{\Sigma }(x,s))_{s\in \omega }$ diverges for each *x*. For the set *B*, the corresponding sequence *converges* on each input *x*—yet, there is no single upper bound *n* on the number of mind-changes required for approximating *all* inputs. Hence, it is reasonable to relax the definition of **FEH** so to include *B*. In the crisp world, sets like *B* are called $\omega$-*computably enumerable* (or $\omega$-c.e.), and they represent the first *transfinite* level of the Classical Ershov Hierarchy. Similarly, we define the $\omega$-level of **FEH** as follows:

#### Definition 8

A fuzzy set *A* is $\omega$-*c.e.* if there exist computable functions $g:\omega \rightarrow \omega$ and $f:\omega \times \omega \rightarrow [0,1]_{\mathbb {Q}}$ such that, for all $x\in \omega$, we have:

- $$\lim _{s\rightarrow \infty } f(x,s)=A(x)$$;
- $$f(x,0) = 0$$;
- $$\vert \{ s\in \omega \,:m^f_{\Sigma }(x,s+1) \ne m^f_{\Sigma }(x,s)\}\vert \le g(x)$$.

So, intuitively, a fuzzy set *A* is $\omega$-c.e. if, for each *x*, we can fix a computable bound (given by *g*) to the number of mind-changes required to approximate *x*. Or, in other words, *A*(*x*) acts as an *n*-c.e. fuzzy set, where *n* equals $g(x)+1$.

Next, similarly to Definition 5, one could introduce the notion of a co-$\omega$-c.e. fuzzy set. Here, we witness the first difference between the finite and the transfinite levels of **FEH**. By Proposition 5, we know that, for all *n*, there is a fuzzy set which is co-*n*-c.e. but not *n*-c.e. Moving to the transfinite levels, such a property fails:

#### Proposition 11

Every co-$\omega$-c.e. fuzzy set is $\omega$-c.e.

#### Proof

Let *A* be a fuzzy set which is co-$\omega$-c.e. Then, there must be computable functions $g:\omega \rightarrow \omega$ and $f:\omega \times \omega \rightarrow [0,1]_{\mathbb {Q}}$ such that for all $x\in \omega$:

- $$\lim _{s\rightarrow \infty } f(x,s)=A(x)$$;
- $$f(x,0) = 1$$;
- $$\vert \{ s\in \omega \,:m^f_{\Pi }(x,s+1) \ne m^f_{\Pi }(x,s)\}\vert \le g(x)$$.

We now define two new computable functions $g'(x)$ and $f'(x,s)$ as follows:

$$\begin{aligned}{} & {} g'(x,s)=g(x,s)+1,\\{} & {} f'(x,0)=0 \text{ and } f'(x,s+1)=f(x,s) \end{aligned}$$

It is easy to see that $f'$ is a $\Delta ^0_2$-approximation of *A*. In addition, the functions $g'$ and $f'$ witness that *A* is an $\omega$-c.e. set.

To define *all* transfinite levels of **FEH**, we shall use the standard technology of Kleene’s notations for computable ordinals $\langle \mathcal {O}, <_\mathcal {O} \rangle$. An interested reader is referred to Rogers (1967) for a classical exposition of Kleene’s $\mathcal {O}$. Intuitively, such a system of notations allows to build up ordinals in an effective manner, by providing suitable codes for each ordinal that can be described in a computable way. For our current purposes, it is sufficient to summarize some key features of $\langle \mathcal {O}, <_\mathcal {O} \rangle$:

1. $\mathcal {O} \subset \omega$, and $\le _{\mathcal {O}}$ is a partial order on the set $\mathcal {O}$;
2. If $a\in \mathcal {O}$, then $\vert a\vert _\mathcal {O}$ denotes the countable ordinal having notation *a*;
3. If $a<_\mathcal {O} b$, then $\vert a\vert _\mathcal {O}<\vert b\vert _\mathcal {O}$ (with respect to the standard order on ordinals);
4. For each $a\in \mathcal {O}$, the (crisp) set $\{ b\in \mathcal {O} \,:b<_{\mathcal {O}} a\}$ is c.e.;
5. There are no infinite decreasing sequences in the poset $\langle \mathcal {O}, <_\mathcal {O} \rangle$;
6. Every finite ordinal *n* has a unique Kleene’s notation.

For the sake of exposition, we start by recalling the definition of the transfinite levels of the Classical Ershov Hierarchy. Note that different authors use slight variations of the following definition, and thus one has to be careful about which type of terminology is used when certain results are stated.

#### Definition 9

Let $a\in \mathcal {O}$ be a notation of a non-zero ordinal. A crisp set $A\subseteq \omega$ *belongs to the class* $\Sigma ^{-1}_a$ (correspondingly, $\Pi _a^{-1}$) if there are computable functions $f:\omega \times \omega \rightarrow \{ 0,1\}$ and $h:\omega \times \omega \rightarrow \{ b\in \mathcal {O} \,:b<_{\mathcal {O}} a\}$ such that for all *x* and *s*:

- $$f(x,0)=0$$ (correspondingly, $$f(x,0)=1$$);
- $$\lim _{s\rightarrow \infty } f(x,s) = A(x)$$;
- $$h(x,s+1) \le _{\mathcal {O}} h(x,s)$$; and
- if $$f(x,s+1) \ne f(x,s)$$, then $$h(x,s+1) \ne h(x,s)$$.

Ershov (1970) proved that the transfinite levels of the Classical Ershov Hierarchy do not collapse. It is now time to define the classes $\Sigma _a^{-1}$ and $\Pi _a^{-1}$ of **FEH**.

#### Definition 10

Let $a\in \mathcal {O}$ be a notation of a non-zero ordinal. A $\Delta ^0_2$ fuzzy set *A* *belongs to the class* $\Sigma ^{-1}_a$ (correspondingly, $\Pi ^{-1}_a$) if there exist a $\Delta ^0_2$-approximation *f*(*x*, *s*) and a computable “counting” function $h:\omega ^2\rightarrow \{ b\in \mathcal {O} \,:b<_{\mathcal {O}} a\}$ such that for all *x* and *s*:

- $$f(x,0) = 0$$ (correspondingly, $$f(x,0)=1$$) and $$\lim _{s\rightarrow \infty } f(x,s) = A(x)$$;
- $$h(x,s+1) \le _{\mathcal {O}} h(x,s)$$;
- if $m^f_{\Sigma }(x,s+1) \ne m^f_{\Sigma }(x,s)$ (correspondingly, $m^f_{\Pi }(x,s+1) \ne m^f_{\Pi }(x,s)$), then $h(x,s+1) \ne h(x,s)$.

Note that the above definition encompasses all finite levels of **FEH**: indeed, if *a* is the notation for a finite ordinal $n\ge 1$, then the class of $\Sigma ^{-1}_a$ sets coincides with the *n*-c.e. fuzzy sets. Moreover, by reasoning as in Proposition 11, it is not hard to see that the classes $\Sigma _a^{-1}$ and $\Pi _a^{-1}$ coincide when *a* is a notation for a limit ordinal.

To gently guide the reader through the proposed transfinite hierarchy, let us discuss a fairly concrete example. Say that *a* is a Kleene’s notation for the ordinal $\omega +\omega +1$. In fact, for the sake of simplicity, let us freely identify notations with the ordinals that they denote. Now, let *A* be a $\Sigma _a^{-1}$ fuzzy set, having a $\Delta _2^0$-approximation *f*(*x*, *s*) and a counting function *h*(*x*, *s*). Then, for a given $x\in \omega$, the behavior of *f*(*x*, *s*) and *h*(*x*, *s*) may look like this:

- At first, the approximating function *f* increases and the counting function *h* just outputs $\omega +\omega$ (note that *h* is required to change its mind only if *f* changes monotonicity). Hence, we cannot predict, or even bound, the number of future mind-changes;
- Next, when *f* changes monotonicity and it starts to decrease, the counting function *f* must update its output to some ordinal strictly less than $\omega +\omega$, that is, to $\omega +n$ for some natural number *n*;
- For the next *n* many mind-changes, our approximation may work as in the $(n+1)$-c.e. case, that is, changing its monotonicity at most *n* many times;
- When the counting function becomes equal to $\omega$, we cannot predict the remaining number of mind-changes;
- The final block of mind-changes works as follows: The approximation changes monotonicity, forcing the value of *h* to decrease to some finite ordinal *k*; next, the approximation works as for a standard $(k+1)$-c.e. or co-$(k+1)$-c.e. set (depending on whether the approximation was increasing or decreasing in the previous step).

More generally, for a fixed transfinite level of **FEH**, the approximation of any given input *x* can always be represented as a finite sequence of blocks. This is because the counting function is non-increasing and there are no infinite decreasing sequences of notations. Each block works as an *n*-c.e. or a co-*n*-c.e. fuzzy set. A priori, we cannot predict either the number of blocks or the size of each of them.

Interestingly, the ordinal $\omega ^2+1$ is able to encode any finite sequence of blocks (of arbitrary but finite size), and thus it can bound the behavior of *any* approximation lying at a given transfinite level of **FEH**. Indeed, let $a\in \mathcal {O}$ be an ordinal notation for $\omega ^2+1$. The counting function for a $\Sigma ^{-1}_a$ fuzzy set starts with output $\omega ^2$. Then, it decreases its value to an ordinal stictly below $\omega ^2$, that is, to an ordinal of the form $\omega \times m+n$, which corresponds to *m* many blocks of unknown size. This idea leads us to the following result:

#### Theorem 12

For a $\Delta ^0_2$ fuzzy set *A*, the following are equivalent:

1. for some $a\in \mathcal {O}$, the set *A* belongs to the class $\Sigma ^{-1}_a$;
2. there exists $b\in \mathcal {O}$ such that $\vert b\vert _{\mathcal {O}} = \omega ^2 + 1$ and *A* belongs to the class $\Sigma ^{-1}_b$.

The proof can be obtained by straightforward computability-theoretic methods, from a similar crisp theorem of Ershov (1970). For reasons of space, we omit the formal construction. On the other hand, by reasoning as in Proposition 5, one obtains that the transfinite hierarchy does not collapse:

#### Proposition 13

Let $a\in \mathcal {O}$ be a notation of a non-zero ordinal, and *A* be a crisp subset of $\omega$. Then *A* is in $\Sigma _a^{-1}$ in the Classical Ershov Hierarchy if and only if *A* belongs to the class $\Sigma _a^{-1}$ in **FEH**. The same is true for $\Pi _a^{-1}$ sets.

The proof is a direct generalization of the proof of Proposition 5: it is enough to replace *n* (which bounds the number of mind-changes) with the counting functions of Definitions 9 and 10.

As a concluding remark, we note that the classes $\Sigma ^{-1}_a$, $a\in \mathcal {O}$, still *do not* exhaust all $\Delta ^0_2$ fuzzy sets. In fact, the same example given in the proof of Proposition 8 applies to the transfinite case: the diagonalization is successful because, for any fuzzy $\Sigma _a^{-1}$ set *A* and any $x\in \omega$, the corresponding $\Sigma$-mind-change sequence $(m^{f}_{\Sigma }(x,s))_{s\in \omega }$ must *converge*, since there are no infinite decreasing sequences in ordinal notations.

## Conclusions

A major goal of this paper has been to highlight the fascinating interplay between two formal approaches to the broad concept of “approximation”. That is, we have combined fuzzy set theory, which provides a mathematical foundation to the idea of *approximate* reasoning, and computability theory, which provides powerful tools to deal with information *approximated* by stages. Building on previous work (Biacino & Gerla, 1989; Gerla, 2001; Harkleroad, 1984, 1988), we introduced and explored the Fuzzy Ershov Hierarchy (**FEH**), which allows to precisely measure how hard it is to approximate certain fuzzy sets. We exhibited both analogies and disanalogies between the Classical Ershov Hierarchy and its fuzzification.

Let us conclude by suggesting two natural ways of expanding our work, which may motivate further research:

1. First, in Sect. 4, we have kept separated the analysis of the refined hierarchy and the transfinite one. Yet, nothing forbids to merge these hierarchies and, e.g., to stratify a given $\Sigma ^{-1}_a$ class of fuzzy sets by keeping track of all updates made by a $\Delta ^0_2$-approximation. It is reasonable to expect that by pursuing such a line of research one will encounter several combinatorial intricacies;
2. Second, it is known that every $\Delta ^0_2$ crisp set belongs to some transfinite level of the Classical Ershov hierarchy (see Ershov (1968b), Theorem 6). As aforementioned, no analogous result holds for fuzzy sets. Hence, it is natural to ask whether there is a natural way of extending **FEH**, beyond the boundaries of Definition 10, so to encompass all $\Delta ^0_2$ fuzzy sets.
